# Supplementary material for: Neurofeedback in ADHD: A qualitative study of strategy use in slow cortical potential training
Source: PLoS One. 2020 Jun 4;15(6):e0233343. doi: 10.1371/journal.pone.0233343 (PMC7272030; doi:10.1371/journal.pone.0233343)
Supplement: S1 Table — Frequency per theme & sub-theme over time, per session. (DOCX) [file pone.0233343.s001.docx]

| **S1 Table.** List of all Domains/Themes/sub-themes. | | | | | |  | |  | |
| --- | --- | --- | --- | --- | --- | --- | --- | --- | --- |
| *Frequency per theme & sub-theme over time, per session.* | | | | | | | |  | |
|  |  |  | |  | |  | |  | |
|  | Strategy | **Session  1** | **Session  5** | **Session  10** | **Session  15** | **Session  20** | **Session  25** | **Session  boosters** |  |
| **C** | **Cognitive** | *57%* | 58% | *70%* | 64% | *81%* | 68% | 56% | D |
| C1 | Focus | *10%* | 17% | *35%* | 29% | *44%* | 37% | 31% | T |
| *C1.1* | *Concentration* | *10%* | 8% | *15%* | 0% | *19%* | 16% | 25% | S |
| *C1.2* | *Directed Focus* | *5%* | 4% | *25%* | 29% | *31%* | 26% | 6% | S |
| *C1.3* | *Scattered Focus* | *5%* | 8% | *10%* | 7% | *0%* | 0% | 0% | S |
| C2 | GIP | *33%* | 33% | *25%* | 29% | *44%* | 42% | 25% | T |
| *C2.1* | *Auditory Imagery* | *14%* | 21% | *5%* | 21% | *19%* | 21% | 6% | S |
| *C2.2* | *Thinking in a Direction* | *14%* | 8% | *10%* | 7% | *19%* | 21% | 13% | S |
| *C2.3* | *Visualization* | *5%* | 8% | *10%* | 14% | *25%* | 5% | 6% | S |
| C3 | Memory Recall | *0%* | 4% | *5%* | 7% | *6%* | 5% | 0% | T |
| C4 | Motivation | *5%* | 0% | *5%* | 7% | *6%* | 5% | 13% | T |
| C5 | Thought Avoidance | *5%* | 8% | *15%* | 14% | *13%* | 5% | 19% | T |
| C6 | Wakefulness | *10%* | 21% | *25%* | 36% | *19%* | 11% | 13% | T |
| *C6.1* | *Alertness* | *10%* | 17% | *10%* | 21% | *13%* | 5% | 6% | S |
| *C6.2* | *Sluggishness* | *5%* | 13% | *20%* | 29% | *13%* | 11% | 6% | S |
| **E** | **Emotional Regulation** | *24%* | 29% | *35%* | 36% | *25%* | 37% | 38% | D |
| E1 | EET | *5%* | 4% | *5%* | 14% | *6%* | 0% | 19% | T |
| E2 | Mindfulness | *14%* | 17% | *20%* | 14% | *13%* | 21% | 19% | T |
| E3 | Specific Emotions | *10%* | 8% | *15%* | 14% | *6%* | 21% | 13% | T |
| **P** | **Physiological** | *24%* | 29% | *25%* | 21% | *31%* | 42% | 56% | D |
| P1 | Breathing | *5%* | 13% | *0%* | 0% | *0%* | 0% | 0% | T |
| P2 | Decreased Activity | *5%* | 8% | *5%* | 0% | *25%* | 32% | 31% | T |
| *P2.1* | *Relaxation* | *5%* | 0% | *5%* | 0% | *13%* | 26% | 25% | S |
| *P2.2* | *Sitting still* | *0%* | 8% | *0%* | 0% | *19%* | 11% | 6% | S |
| P3 | Muscular activity | *24%* | 13% | *20%* | 21% | *6%* | 16% | 31% | T |
| **U** | **Unspecified** | *38%* | 50% | *65%* | 36% | *38%* | 42% | 44% | D |
| U1 | Disengaged | *10%* | 0% | *5%* | 14% | *13%* | 21% | 6% | T |
| U2 | Experimenting | *14%* | 21% | *5%* | 7% | *6%* | 5% | 13% | T |
| U3 | Lack of Insight | *10%* | 13% | *25%* | 0% | *13%* | 5% | 25% | T |
| U4 | Passivity | *14%* | 25% | *35%* | 21% | *6%* | 32% | 31% | T |
|  |  |  |  |  |  |  |  |  |  |
|  |  |  |  |  |  |  |  |  |  |
| *Note.* GIP= Generating Internal Phenomena, EET= Emotionally Engaging Thought  D= Domain, T= Theme, S= Sub-theme | | | | | | | | | |
