# Supplementary figures and images for: Neurofeedback in ADHD: A qualitative study of strategy use in slow cortical potential training
Source: PLoS One. 2020 Jun 4;15(6):e0233343. doi: 10.1371/journal.pone.0233343 (PMC7272030; doi:10.1371/journal.pone.0233343)

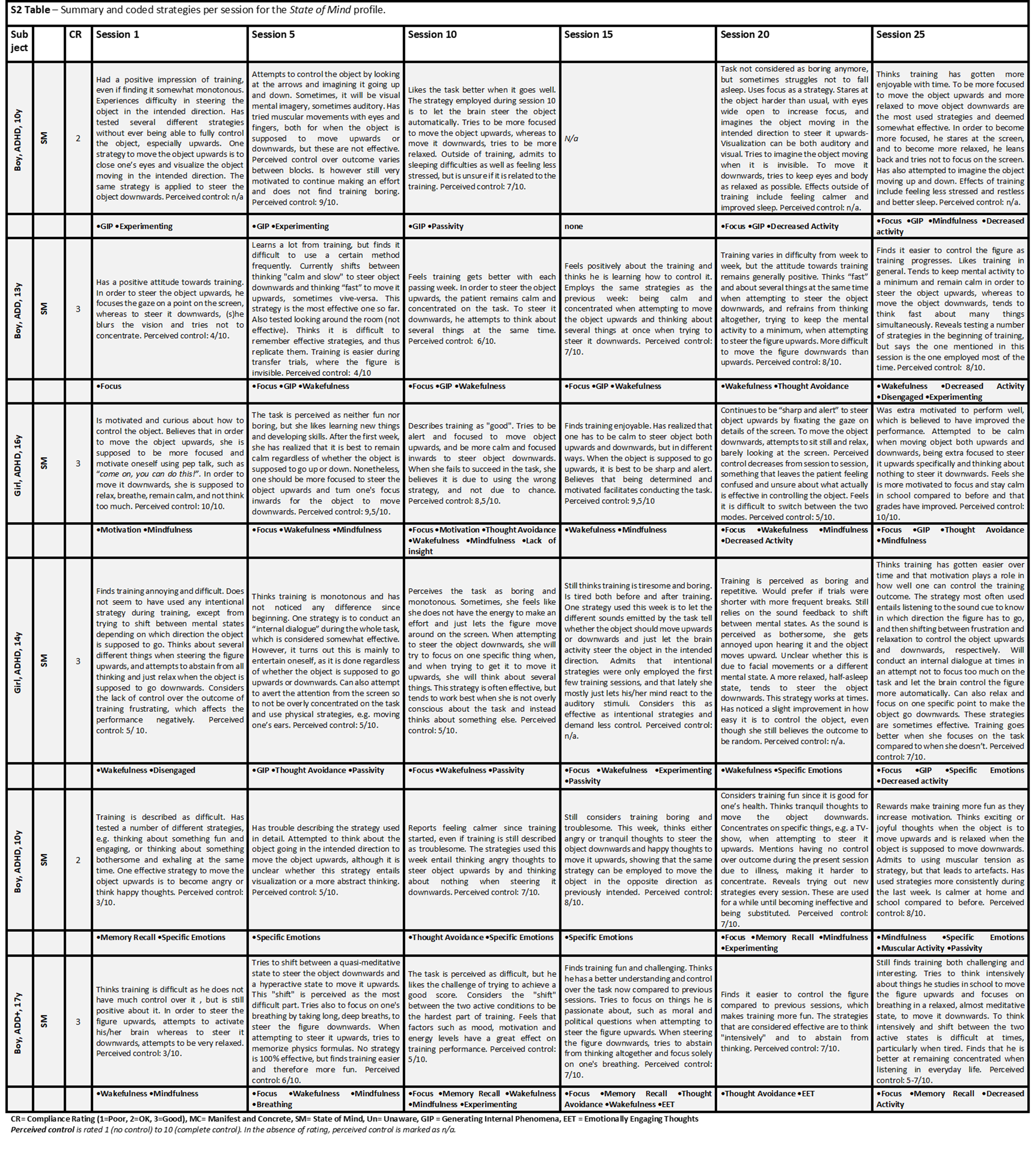

Supplement: S2 Table — Session summaries of each individual interview that is included in the strategy profile. (PNG) [file pone.0233343.s002.png]

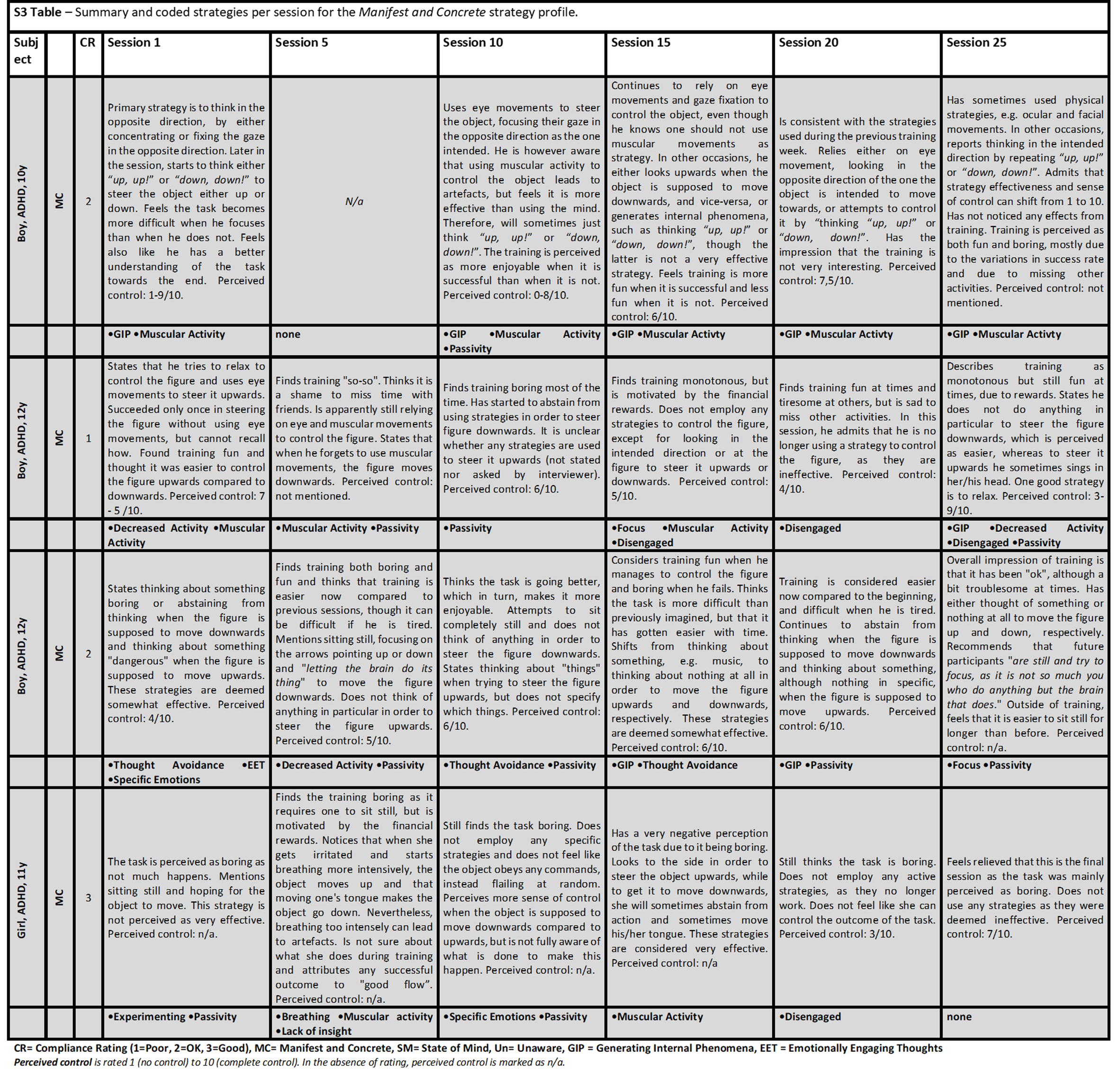

Supplement: S3 Table — (PNG) [file pone.0233343.s003.png]

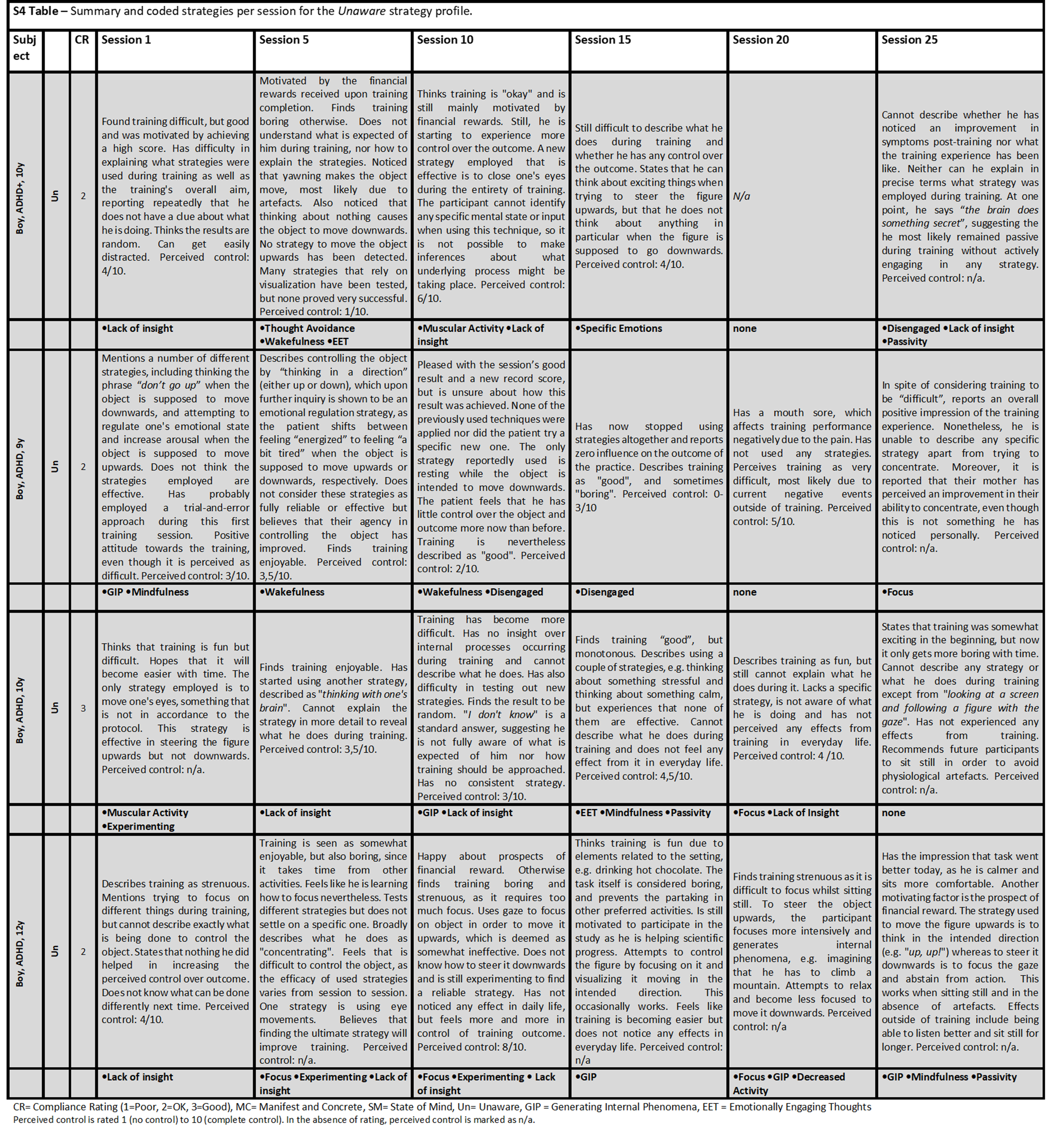

Supplement: S4 Table — Session summaries of each individual interview that is included in the strategy profile. (PNG) [file pone.0233343.s004.png]
