# Supplementary material for: Neurofeedback in ADHD: A qualitative study of strategy use in slow cortical potential training
Source: PLoS One. 2020 Jun 4;15(6):e0233343. doi: 10.1371/journal.pone.0233343 (PMC7272030; doi:10.1371/journal.pone.0233343)
Supplement: Attachment — Submitted filename: Respondse to Reviewers.docx [file pone.0233343.s006.docx]

| Reviewer #1: The authors present a very interesting qualitative study into strategy use during SCP neurofeedback in ADHD. I agree that these kind of studies are important and long overdue, and I am hopeful that these insights will contribute to a better and more nuanced understanding of neurofeedback as treatment for ADHD. The manuscript is well written and the methods seem sound, I mostly have minor comments and ideas for additional discussion. | Thank you very much, we truly appreciate your recognition of the importance of our research. |
| --- | --- |
| Introduction |  |
| 1. In the explanation of neurofeedback (lines 75-77), you may add that other important ‘ingredients’ are learning principles, such as operant conditioning. | Thank you, other learning principles have been added to the introduction. |
| 2. The first sentence of the paragraph (starting at line 90) does not really represent the rest of the paragraph, which mostly sums up the many limitations in neurofeedback research. | Thank you for addressing this, we have rewritten the opening sentence for clarity. |
| 3. When discussing specific versus non-specific effects, which is at the core of the neurofeedback efficacy ‘debate’, you may want to consult the CRED-nf checklist. Recently, a consensus report was drafted on the reporting and experimental design of neurofeedback studies. The report contains a useful model that distinguishes multiple mechanisms that together may drive the total effect of neurofeedback, including (1) neurofeedback specific, (2) neurofeedback non-specific, (3) general non-specific, (4) repetition-related and (5) natural mechanisms. | Thank you very much for bringing this to our attention. We have completed the section where we discussed specific versus non-specific effects, to also mention repetition-related and natural mechanisms. We now also cite the CRED-nf checklist here, which is an excellent reference in the given context |
| Methods |  |
| 1. Although the authors provide more details on the specific instructions that participants received during the neurofeedback session in the discussion, please also provide those in the methods section (were there any instructions in the protocol?): (a) How was the SCP training framed? As a way to treat ADHD, to self-regulate attention?, (b) How were the participants instructed concerning physiological artefacts?, (c) What instructions did they receive during the sessions? | Thank you for this very valuable request for clarification regarding the points a, b, and c. We have elaborated the “SCP task” section, and added information addressing the framing of the training and the instructions that were given to the participants. |
| 2. On lines 241-242, it is described that mostly successful strategies were explored. Although this makes sense, do the authors think future research should also ask about what strategies did not work, and how many strategies were used during each session (as a measure of trial-and-error learning)? | Thank you and apologies for the lack of clarity. Non-effective strategies were also addressed by some participants, when we followed up the participants’ answers with the standard question: “*how did that work for you*”. Thus, both successful and less successful strategies experienced by the participants were assessed. Nonetheless, a more focused, elaborated and explicit inquiry about the non-successful strategies would surely have been desirable and should be included in future research.  The number of strategies reported in each interview is shown in the supporting information Tables S2-S4. |
| Results |  |
| 1. I think most results are presented well, but there is some inconsistent use of the words ‘theme’ and ‘sub-theme’ throughout the results section, and Table 2 (which ones are italic for example), making it more difficult to follow. | Thank you for pointing this out to us. There were some inconsistencies in using *italics* that have been corrected. In addition, we added a legend to table 2 and 4 to further clarify the themes and subthemes. |
| 2. It may be useful to provide a ‘overview’ figure with all domains, and the themes and subthemes (this could be depicted using hierarchical boxes on top of each other). | We agree that a figure showing the pattern of themes and subthemes may be useful, and have added a figure illustrating the theme structure early in the result section. |
| 3. Line 373: also name the number of themes and subthemes, including their names (like the other domain paragraphs). | Done. |
| 4. I believe the subtheme ‘silence’ is not discussed in the text (of physiological strategies), while ‘breathing’ twice. | True - thank you for noticing. *Silence* was a “isolated” subtheme and only defined by one quote. It was removed due to its limited significance, and its defining statement was later moved to “Passivity” as the essence was to be quiet. We had removed it from the text but had missed removing it from the table, which now has been done. |
| Discussion |  |
| 1. It is fascinating to recognize many strategies, as reported in this manuscript, in my own experience with theta/beta neurofeedback research in ADHD and conversations I had with participants. Although SCP and theta/beta neurofeedback have different targets, both seem to affect (or regulate, in SCP) arousal. It may be interesting to discuss what the results mean for other neurofeedback protocols. | Very interesting to hear that the reviewer shares our experience regarding the strategies for T/B NF. In the KITE-study, we have also gathered qualitative data on strategies using interviews for live z-score NF. Initially, we had planned to compare them with SCP in one paper, but we had to cancel that plan since the amount of information would have been to extensive to wrap in one journal manuscript. Instead the strategies used in z-score training will be presented in a separate later paper. Still, in order to address the reviewer’s valid point, we have added some lines on this topic to the paper’s discussion sections. |
| 2. The authors discuss three different strategy ‘profiles’. The ‘state of mind’ profile is associated with intrinsic motivation, which may be an important factor in the ultimate efficacy of the training (although this is not studied in the current manuscript). It would be very interesting if the authors discuss the role of motivation more extensively. For example, the role of self-efficacy, locus-of-control, etc. in ADHD and as potential moderator for strategy use and efficacy of neurofeedback in ADHD. | We agree completely, that these are very relevant suggestions, why we have extended the discussion of motivation as recommended in the respective section. Furthermore, we will address these questions more thoroughly in a future paper that is in progress. |
| 3. Would you recommend exploring strategy use in EMG biofeedback (as sham-control condition for neurofeedback)? It may be interesting to see whether there is an overlap in strategies used, which may explain non-specific effects of neurofeedback. | Thank you for your input. We agree that it would be especially interesting to explore strategies in sham-control conditions, considering that they often also lead to symptom improvements. Accordingly, this aspect has been added to the discussion section. |
| 4. Line 729: typo (the word ‘not’ is missing between ‘did’ and ‘benefit’).  Tieme Janssen | Corrected, thank you, very attentive of you. |
|  |  |
|  |  |
| Reviewer #2: The manuscript entitled „Neurofeedback in ADHD: a qualitative study of strategy use in slow cortical potential training“ reports on the results of a qualitative explorative investigation of strategies during SCP-NF in 30 children and adolescents with ADHD. The authors derived strategy clusters covering cognitive, physiological, emotional and unspecified domains. Furthermore, from 14 participants, three strategy profiles could be differentiated: “State of Mind”, “Manifest and Concrete” and “Unaware”. This is an interesting study with the rationale to shed light on NF mechanisms. The manuscript is well written and clearly structured, and I believe that the results are of interest to other researchers in the field. | Thank you for all the positive and encouraging comments. |
| However, it would seem important to link the qualitative data (/subjective experience) at least to SCP regulation performance (i.e. amplitudes as an objective measure). The mere description of strategies doesn’t help evaluating how efficacious they are on NF performance. Thus, the final conclusion that the findings may help to facilitate more effective instructions in how to approach SCP is not supported by the data. From the data provided in the current version of the manuscript, we can’t see which strategies avail or hinder treatment effects or if using explicit strategies would be useful at all. Thus, we learn from the manuscript which strategies are used, but we do not know which of them are advantageous. | Yes, this is a very important point you make, and we have added an exploratory analysis of the SCP regulation performance for the three types of strategy profiles.  A further analysis of the impact of individual strategies on regulatory performance would be very interesting for future studies, with the current data-set, however, it is not possible. |
| I believe that it would increase the informative value for the scientific community tremendously if the authors would build the bridge between the strategies or the strategy profiles and NF performance (cf., e.g., Kober et al., 2013) as well as symptom changes. This is, in my opinion, the most important part as it gives information on:  - Which strategies/ strategy profiles are (not) effective ((not) leading to negative or positive SCP shifts)? Can strategies predict outcome (Conners)?  - Are there differences in which strategies are particularly effective/feasible in everyday life (leading to more pronounced changes in Conners)?  o e.g., negative emotional strategies (anger, sadness) might be less feasible in everyday life as inducing negative emotions to take yourself into a state of attention might not be the appropriate approach  - Are there differences in which strategies are particularly effective depending on presentations of ADHD (Conners subscales)?  o e.g., the physiological strategies most probably constitute a general physical arousal regulation rather than regulation of a circumscribed disorder-specific brain region (thereby leading to specific ADHD-related symptom changes). Still, they might be particularly effective in reducing motor hyperactivity (by general relaxation).  - Is lack of insight an advantage (leading to more pronounced amplitudes and speaking in favor of an automatic learning process)? | We agree that linking the different elements of 1) strategy use, 2) NF performance (self-regulation) and 3) symptom outcomes is highly significant.  Please notice, however, that the nature of this study is qualitative (!) and exploratory, more generating hypotheses, than testing any with regard to strategy use. The data that the reviewer is requesting would rather be meaningful to be collected in an follow-up study based on the current findings. Nonetheless, we have now added data to the paper linking strategies and performance as much as possible and reasonable. We hope the reviewer finds it informative that we provide self-regulation performance in SCP for the three derived strategy profiles. However, this information is also of rather exploratory and preliminary nature. |
| Minor: |  |
| Reports from teachers or parents occurred by report of the children?  How did these reports coincide with the ratings by teachers or parents themselves? | Yes, all statements are based on the interviews with the children and adolescents. |
| In Figure 1 SCP Feedback. Looking at the feedback screen depicted in the middle, it looks as if a threshold would have been implemented during training, which is not mentioned in the manuscript and supposedly was not the case. | Correct, with few exceptions there was a threshold implemented during the training. We have clarified this in the section addressing the “SCP task”. |
| The authors should recheck spaces (e.g. line 50, 56, 94, 254) and misplaced words (e.g., line 152, 603).  In line 530, it should read “(…) frequently, showing that many strategies (…)” | Thank you, very attentive of you. It has been corrected. |
|  |  |
| Reviewer #3: In this manuscript, the authors analyzed quantitatively [editor: I think they meant qualitatively] the self-regulation strategies during specific and standardized neurofeedback training in ADHD patients. This is an important study which adds relevant and highly novel insights to the field of neurofeedback training. The main aims were to map the cognitive strategies, evaluate the changes over time and to investigate different profiles. | We are grateful for your recognition of our study. |
| The manuscript is well written, but the result section is difficult to follow. In my opinion, it would be helpful to include some tables in which the proportion of subjects and the different strategies are stated and classified. | We apologize for the lack of clarity. Table S1 in the supporting information lists all strategies (themes and sub-themes) including the frequency per session. We had this table in the main text in earlier drafts of the manuscript. We have also added an overview figure, which we hope will help. |
| One major concern needs to be addressed to be suitable for publication and therefore to add novel insights to the field. In my opinion, the authors must include the factual self-regulation data of the NF training and performance. This is important to be able to answer the question of which cognitive or emotional strategy worked. Now the manuscript only is able to show in a quantitatively [editor: I think they mean qualitatively here] way that the participants tried something, but the key question remains unanswered! Did a specific strategy work? Did one work better? – In my opinion, this is really important and would lead to a very important paper. The authors might just look at the mean % of suns delivered by Theraprax?  If the self-regulation data is not included, the authors should address this issue in more detail during the discussion or even add a limitation section. | Please see also our more extensive reply to reviewer #2.  Thank you – the reviewer raises of course relevant questions. To comply with these we have added information on the SCP regulation performance for the different derived strategy profiles.  Nonetheless, please note that this study is a qualitative one, of exploratory nature and personal experiencing, rather generating hypotheses of used strategies, than testing the effects of established strategies. |
| Minor: |  |
| Introduction: |  |
| L70 Citation error | Thank you, this has been corrected. |
| L81 ADHD symptoms as well as for medical conditions the authors may add a citation | Thank you, the sentence has been revised. |
| L93 – The authors should cite more recent meta-analysis (ie. Cortese et al., 2016, Riesco-Mejias et al 2019) | Thank you very much for bringing this to our attention. We have included the Riesco-Matías et al meta-analysis. |
| L128 The authors should consider as well following manuscript when addressing the Placebo question in NF (Busalb et al 2019 https://www.frontiersin.org/articles/10.3389/fpsyt.2019.00035/full) | Thank you for bringing the Bussalb et al article to our attention. We have incorporated it in the text. |
| L131 The authors should specify that this study was done in Adults | Thank you, we have clarified this. |
| Methods: |  |
| Discussion: |  |
| L 629 The authors state that they do not know if the subjects succeed in modulating their brain activity. This is a critical point and in my point of view if included it will enrich the manuscript and the conclusions. | Agreed; this is indeed highly relevant. Though we pursue an exploratory, qualitative approach, we have added a brief examination of the self-regulation performance during SCP for the three derived strategy profile groups. |
| Did the participants receive some instruction prior to the SCP-NF training? | Thank you for pointing this out. We have elaborated the instructions given to the participants in the method. |
| I miss the differentiation between strategies for the different conditions. SCP-NF has two conditions which are Negativity and Positivity trials. Did the authors look if there were different strategies during those different conditions? - What about the transfer vs. real-time feedback? | Yes, this was something that we addressed in our initial analysis phase, when the verbal material was structured based on the conditions. Still we merged the material again subsequently, as many strategies, for both Negativity and Positivity, are simply inverted versions dependent on the direction.  In addition, as for transfer/real-time feedback, the extent and depth of the responses given by the child and adolescent participants was not rich enough to go into such detail in a meaningful way. Instead, we focused on broader themes that are describing strategies used for all conditions. |
| L634 “While the other two groups also improvements were reported” I think that here is missing something. | Yes, it should have read: “*While the other two groups also reported improvements,…*”. This has been corrected. |
| L717 though we do not know whether these individuals were successful in manipulating their slow cortical potentials. The authors must analyse that. | See also our responses above. We hope that the newly added data on the SCP regulation addresses this issue sufficiently. Again, this is predominately a qualitative study. |
|  |  |
